# Supplementary material for: Comprehensive Analysis and Drug Modulation of Human Endogenous Retrovirus in Hepatocellular Carcinomas
Source: Cancers (Basel). 2023 Jul 18;15(14):3664. doi: 10.3390/cancers15143664 (PMC10377948; doi:10.3390/cancers15143664)
Supplement: Supplementary file 1 [file cancers-15-03664-s001.zip › Supplementary figures.pdf]

Supplementary Figures

Figure S1. The distributions of differentially expressed HERVs in the four HCC subgroups.

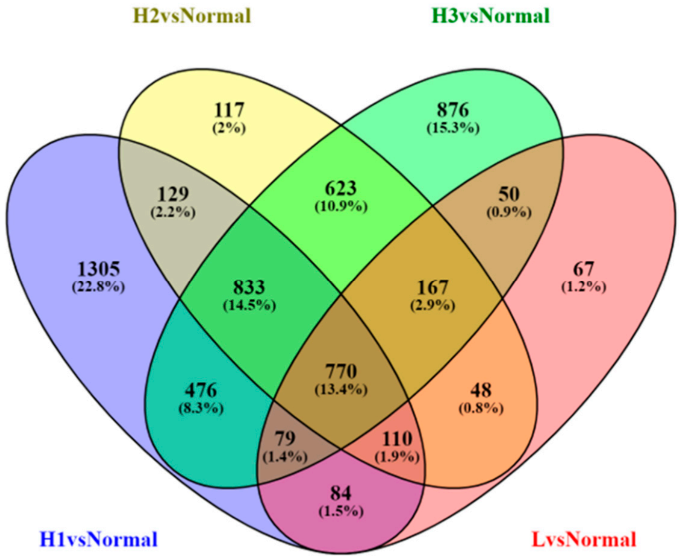

Figure S2. Association between HCC subgroups and mutations.

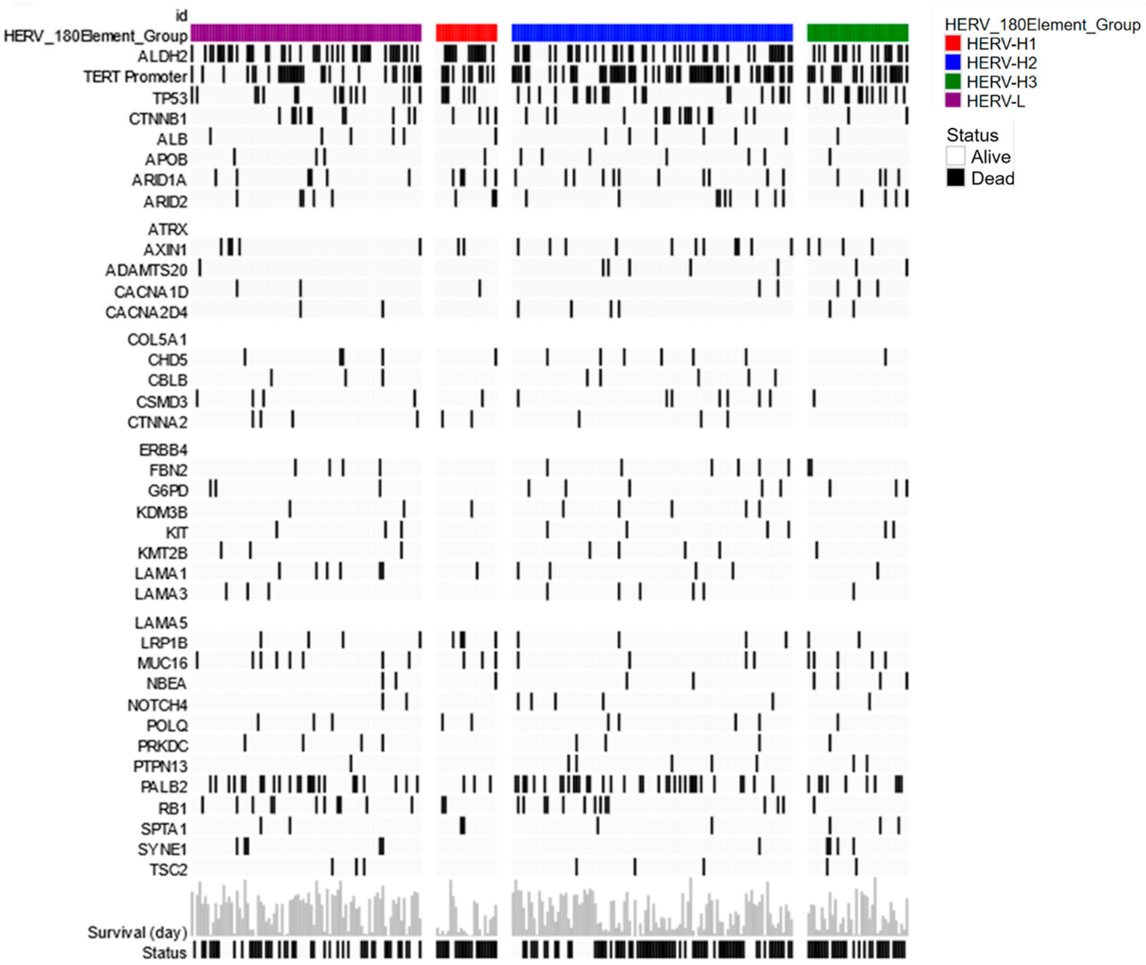

Figure S3. Association between HCC subgroups and copy number changes.

A

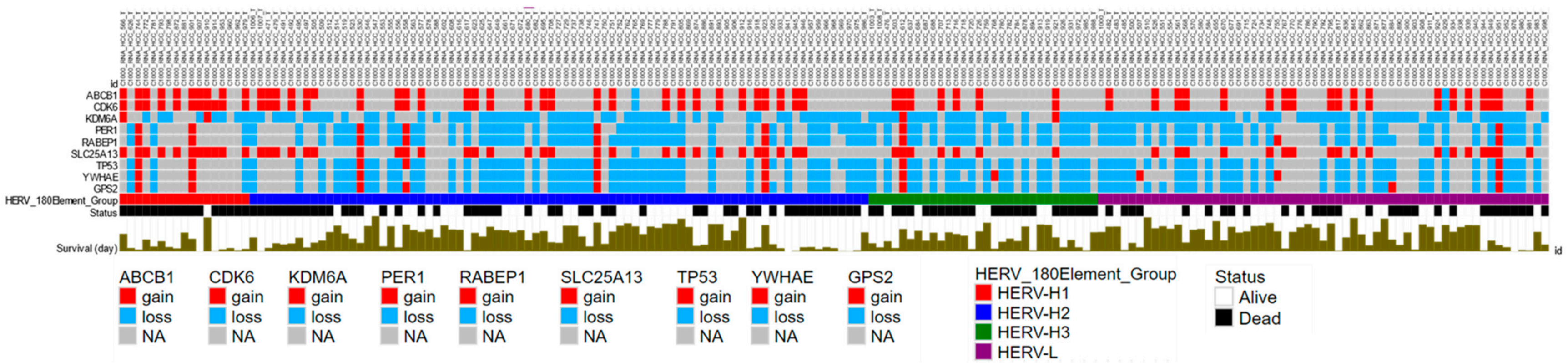

B

	level	Overall	HERV-H1	HERV-H2	p
KDM6A (%)	gain	2 (3.0)	2 (20.0)	0 (0.0)	0.017
	loss	64 (97.0)	8 (80.0)	56 (100.0)	
	level	Overall	HERV-H1	HERV-L	p
KDM6A (%)	gain	2 (3.4)	2 (20.0)	0 (0.0)	0.028
	loss	56 (96.6)	8 (80.0)	48 (100.0)	
TP53 (%)	gain	3 (7.9)	2 (40.0)	1 (3.0)	0.049
	loss	35 (92.1)	3 (60.0)	32 (97.0)	

Figure S4. Association between HCC subgroups and structural variants.

A

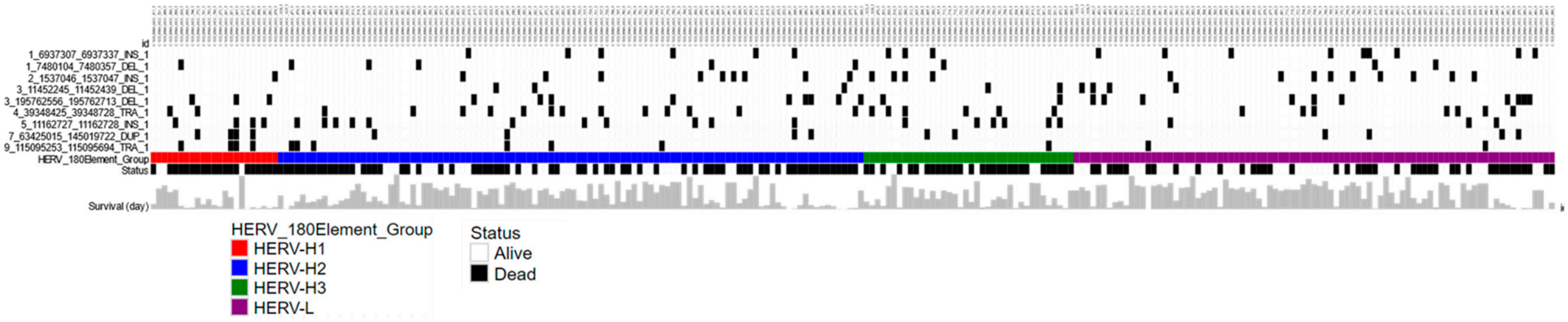

B

	level	Overall	HERV-H1	HERV-H2	p
SV: 7_63425015_145019722_DUP_1	No	121 (93.8)	19 (82.6)	102 (96.2)	0.048
	Yes	8 (6.2)	4 (17.4)	4 (3.8)	
	level	Overall	HERV-H1	HERV-L	p
SV: 9_115095253_115095694_TRA_1	No	104 (94.5)	19 (82.6)	85 (97.7)	0.02
	Yes	6 (5.5)	4 (17.4)	2 (2.3)	
SV: 5_11162727_11162728_INS_1	No	103 (93.6)	19 (82.6)	84 (96.6)	0.05
	Yes	7 (6.4)	4 (17.4)	3 (3.4)	
	level	Overall	HERV-H3	HERV-L	p
SV: 5_11162727_11162728_INS_1	No	116 (92.8)	32 (84.2)	84 (96.6)	0.038
	Yes	9 (7.2)	6 (15.8)	3 (3.4)	
	level	Overall	HERV-H	HERV-L	p
SV: 5_11162727_11162728_INS_1	No	230 (90.6)	146 (87.4)	84 (96.6)	0.033
	Yes	24 (9.4)	21 (12.6)	3 (3.4)	

Figure S5. Classification of TCGA-LIHC using 180 HERVs transcriptome. (A) Unsupervised clustering analysis of 180 HERVs for TCGA-LIHC and nearby non-cancerous tissues. Two distinct subgroups (A and B) in the heatmap at cluster. (B) Overall survival of two subgroups.

A

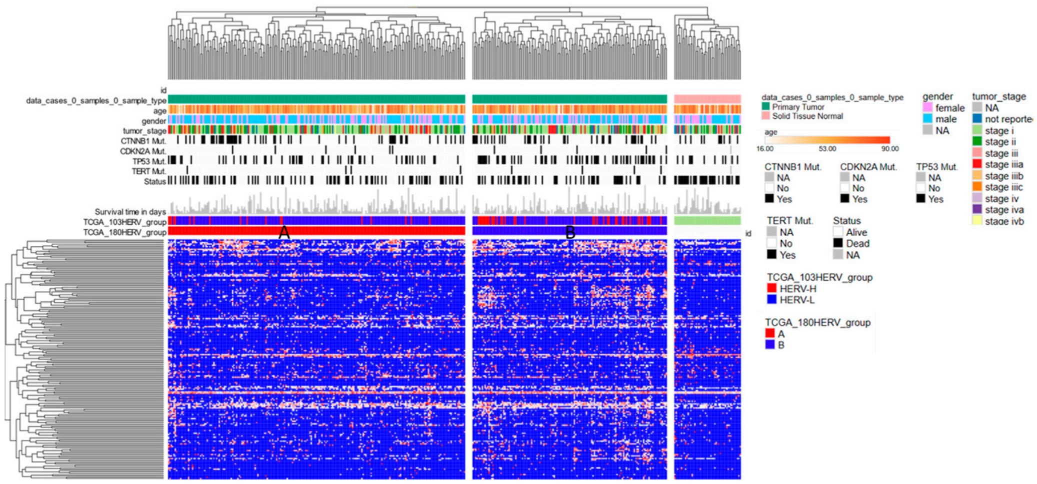

B

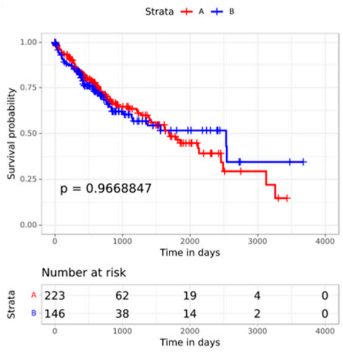

Figure S6. Venn diagrams representing the interrelationships of survival-related differentially expressed HERVs (A) and nearby genes (B) between our cohort and TCGA-LIHC.

A

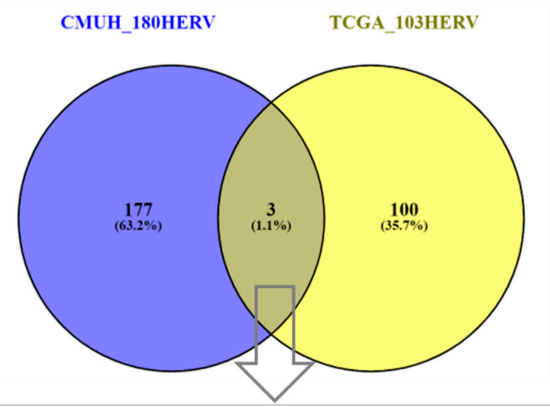

Locid	Seqname	Start	End	Strand	Gene_id	Class	Family
LTR25-int_0094	chr11	129779830	129785817	-	LTR25_11q24.3b	HERV	LTR25
HERV3-int_0065	chr19	566440	568668	-	HERV3_19p13.3	HERV	HERV3
ERVL-B4-int_1835	chrX	86861009	86867126	+	ERVLB4_Xq21.2	HERV	ERVLB4

B

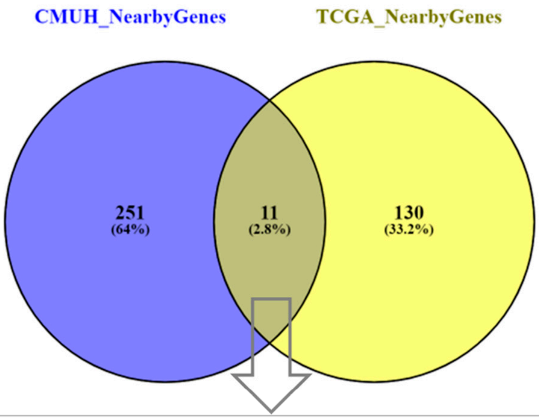

Geneid	Seqname	Gene_type	Gene_name
ENSG00000227471.9	chr7	protein_coding	AKR1B15
ENSG00000267751.5	chr19	lncRNA	AC009005.1
ENSG00000287426.1	chr11	lncRNA	AP003327.2
ENSG00000237653.2	chr3	processed_pseudogene	AC026320.2
ENSG00000279013.1	chr4	TEC	AC110774.1
ENSG00000126733.21	chrX	protein_coding	DACH2
ENSG00000283646.2	chr3	lncRNA	LINC02009
ENSG00000201860.1	chr3	misc_RNA	Y_RNA
ENSG00000249891.2	chr4	processed_pseudogene	KRT19P6
ENSG00000236190.1	chrX	processed_pseudogene	Z82210.1
ENSG00000281566.3	chrX	lncRNA	AL157778.1

Figure S7. The higher expressed genes in the HCC subgroups for the gene panels of HERV activation restriction (A), HERV sequence binding proteins (KZFPs) (B), RNA transport (C), stemness (D), metabolism (E), antiviral immunity (F), human leukocyte antigen (HLA)/antigen presentation (G), immune checkpoint (H), inflammasome and inflammatory response (I), stimulated 3 prime antisense retroviral coding sequences (SPARCS) (J) and nearby genes (K).

A

Gene panel	HERV-H1vs HERV-H2	HERV-H1vs HERV-H3	HERV-H1vs HERV-L	HERV-H2 vs HERV-H3	HERV-H2 vs HERV-L	HERV-H3 vs HERV-L
HERV activation restriction (96)	IGF2BP (H1)	H3-3A (H1)	H3-3A (H1)	DNMT3B (H3)	GNAS (L)	KDM1A (H3)
	H3-3A (H1)			AGO1 (H3)	PIWIL4 (L)	AGO1 (H3)
				FOXM1 (H3)	APOBEC3D (L)	SMUG1 (H3)
				IGF2BP3 (H3)		IGF2BP3 (H3)
				TET1 (H3)		RESF1 (L)
				IGF2BP1 (H3)		
				RESF1 (H2)		

B

Gene panel	HERV-H1vs HERV-H2	HERV-H1vs HERV-H3	HERV-H1vs HERV-L	HERV-H2 vs HERV-H3	HERV-H2 vs HERV-L	HERV-H3 vs HERV-L
KZFPs (101)		ZNF215 (H3)		ZNF215 (H3)	ZNF222 (H3)	ZNF426 (H3)
		ZNF534 (H3)		ZNF382 (H3)	ZFP1 (H2)	ZNF215 (H3)
		ZNF90 (H3)		ZNF496 (H3)		ZNF222 (H3)
		ZNF578 (H3)		ZNF283 (H3)		ZNF496 (H3)
				ZNF354C (H3)		ZNF283 (H3)
				ZNF320 (H3)		ZNF320 (H3)
				ZNF93 (H3)		ZNF93 (H3)
				ZNF793 (H3)		ZNF793 (H3)
				ZNF724 (H3)		ZNF724 (H3)
				ZNF695 (H3)		ZNF695 (H3)
				ZNF534 (H3)		ZNF534 (H3)
				ZNF611 (H3)		ZNF611 (H3)
				ZNF90 (H3)		ZNF90 (H3)
				ZNF736 (H3)		ZNF812P (H3)
				ZNF578 (H3)		ZNF739 (H3)
						ZNF578 (H3)

C

Gene panel	HERV-H1vs HERV-H2	HERV-H1vs HERV-H3	HERV-H1vs HERV-L	HERV-H2 vs HERV-H3	HERV-H2 vs HERV-L	HERV-H3 vs HERV-L
RNA transport (70)	THOC3 (H1)		THOC3 (H1)	THOC3 (H3)		THOC3 (H3)
				NDC1 (H3)		NDC1 (H3)
				THOC5 (H3)		NUP93 (H3)
				NUP93 (H3)		NUP107 (H3)
				NUP107 (H3)		NUP35 (H3)
				SRSF12 (H3)		
				NUP35 (H3)		

D

Gene panel	HERV-H1vs HERV-H2	HERV-H1vs HERV-H3	HERV-H1vs HERV-L	HERV-H2 vs HERV-H3	HERV-H2 vs HERV-L	HERV-H3 vs HERV-L
Stemness (126)	BCKDHB (H2)	BCKDHB (H3)	ERCC2 (H1)	SEMA3A (H3)	EPCAM (L)	SEMA3 (H3)
	BMPR1A (H1)	DTD (H1)	DTD1 (L)	ORC1 (H3)	LIN28B (H2)	ORC1 (H3)
	GBE1 (H2)	RRAS2 (H3)	RRAS2 (L)	DNMT3B (H3)		ERCC2 (H2)
	EPCAM (H1)	INHBE (H3)	INHBE (L)	CENPI (H3)		KIF20A (H3)
	DTD1 (H1)	LIN28B (H3)	ANPEP (L)	KIF20A (H3)		CPSF3 (H3)
	RRAS2 (H2)		ROR1 (H1)	DLGAP5 (H3)		DLGAP5 (H3)
	INHBE (H2)			NREP (H3)		ARMC9 (H3)
	HMGA2 (H1)			ARMC9 (H3)		DIAPH3 (H3)
	IGF2BP1 (H1)			TET1 (H3)		CENPH (H3)
	GPX8 (H1)			DIAPH3 (H3)		FANCB (H3)
	PFAS (H1)			CENPH (H3)		LIN28B (H3)
	ROR1 (H1)			IGF2BP1 (H3)		
				PFAS (H3)		
				FANCB (H3)		
				LIN28B (H3)		

E

Gene panel	HERV-H1vs HERV-H2	HERV-H1vs HERV-H3	HERV-H1vs HERV-L	HERV-H2 vs HERV-H3	HERV-H2 vs HERV-L	HERV-H3 vs HERV-L
Metabolism (51)	ACSM2B (H2)	SLCO1B3 (H1)	KMO (L)	OTC (H2)	TYR03 (L)	ENO1 (H3)
	TYRO3 (H1)	ACADL (H3)	RHBG (H1)	PKM (H3)	PDE9A (L)	MTHFD1 (L)
	PCK2 (H2)	KMO (H3)	CYP2C8 (L)	SLC27A5 (H2)		SLCO1B3 (L)
	SULT22A1 (H2)	RHBG (H1)	CYP11A1 (L)	TYRO3 (H3)		SRM (H3)
	ACADL (H2)	PGD (H3)	TDO2 (L)	MTHFD1 (H2)		CDO1 (L)
	KMO (H2)	XDH (H3)	XDH (L)	PCK2 (H2)		CYP2C8 (L)
	RHBG (H1)			AQP9 (H2)		PGD (H3)
	CYP2C8 (H2)			SULT2A1 (H2)		G6PD (H3)
	DPYS (H2)			IMPDH1 (H3)		CYP3A4 (L)
	TDO2 (H2)			SLC6A12 (H2)		
	XDH (H2)			SLCO1B3 (H2)		
	CYP4A22 (H2)			ALDH6A1 (H2)		
				CDO1 (H2)		
				CYP2C9 (H2)		
				CYP2C8 (H2)		
				AOX1 (H2)		
				SORD (H2)		
				PGD (H3)		
				MAT1A (H2)		
				G6PD (H3)		
				CYP3A4 (H2)		
				NAGS (H2)		

F

Gene panel	HERV-H1vs HERV-H2	HERV-H1vs HERV-H3	HERV-H1vs HERV-L	HERV-H2 vs HERV-H3	HERV-H2 vs HERV-L	HERV-H3 vs HERV-L
Antivival immunity (263)	RPLP0 (H1)	PHLDA1 (H1)	GADD45B (L)	CD276 (H3)	LGALS3BP (L)	SNRNP200 (H3)
	GCH1 (H2)	TRIM5 (H3)	CDKN1A (L)	DHX58 (H2)	IL15RA (H2)	IFIT5 (L)
	APP (H2)	APP (H3)	GCH1 (L)	SNRNP200 (H3)	HERC5 (H2)	IL12A (L)
	ACSL1 (H2)	ACSL1 (H3)	APP (L)	PTGES (H3)	ACSL1 (H2)	
	OLR1 (H1)	ZNF503 (H1)	ACSL1 (L)	RNASE2 (H3)	MAPK13 (L)	
	MARCKSL1 (H1)		ETS2 (L)	IKBKE (H3)	LY6E (L)	
	UBA52 (H1)		MARCKSL1 (H1)		IFITM10 (L)	

G

Gene panel	HERV-H1vs HERV-H2	HERV-H1vs HERV-H3	HERV-H1vs HERV-L	HERV-H2 vs HERV-H3	HERV-H2 vs HERV-L	HERV-H3 vs HERV-L
HLA&antigen presentation (45)		HLA-E (H1)				HLA-E (L)

H

Gene panel	HERV-H1vs HERV-H2	HERV-H1vs HERV-H3	HERV-H1vs HERV-L	HERV-H2 vs HERV-H3	HERV-H2 vs HERV-L	HERV-H3 vs HERV-L
Immune checkpoint (22)	TNFRSF9 (H1)		TNFRSF9 (H1)	CD276 (H3)		

I

Gene panel	HERV-H1vs HERV-H2	HERV-H1vs HERV-H3	HERV-H1vs HERV-L	HERV-H2 vs HERV-H3	HERV-H2 vs HERV-L	HERV-H3 vs HERV-L
Inflammasome and Inflammatory	IL32 (H2)		IL32 (L)	C4BPA (H2)	IL15RA (L)	
	IL4R (H2)		IL4R (L)		SIGIRR (L)	

J

Gene panel	HERV-H1vs HERV-H2	HERV-H1vs HERV-H3	HERV-H1vs HERV-L	HERV-H2 vs HERV-H3	HERV-H2 vs HERV-L	HERV-H3 vs HERV-L
SPARCS (15)	IL32 (H2)		IL32 (L)			
	TNFRSF9 (H1)		TNFRSF9 (H1)			

K

Gene panel	HERV-H1vs HERV-H2	HERV-H1vs HERV-H3	HERV-H1vs HERV-L	HERV-H2 vs HERV-H3	HERV-H2 vs HERV-L	HERV-H3 vs HERV-L
Nearby genes (262)	KAV3 (H2)	AKR1B15 (H3)	NAV3 (L)	ZNF678 (H3)	AC004083.1 (H2)	GABRA2 (H3)
	LINC02819 (H2)	MIR4500HG (H3)	MIR4500HG (H1)	LINC02163 (H3)	GABRA2 (H2)	ZNF221 (H3)
	AKR1B15 (H2)	PORPL (H3)	LINC02163 (H1)	LINC02055 (H3)	LINC02819 (H2)	MIR4500HG (H3)
	PORPL (H2)	AC093916.1 (H3)	AL390334.1 (H1)	AL390334.1 (H3)	MIR4500HG (H2)	LUCAT1 (H3)
	AC093916.1 (H2)	LINC02055 (H3)	RNPS1P1 (H1)	LINC00534 (H3)	POPRL (H2)	LINC01060 (H3)
	DACH2 (H1)	AL390334.1 (H3)	AC006525.1 (H1)	DTL (H3)	LINC02163 (H2)	PURPL (H3)
	AL157778.1 (H2)	KCNH8 (H3)	DTX4 (L)	KCNH8 (H3)	AC093916.1 (H2)	LINC02163 (H3)
	AC026414.1 (H2)	MEI4 (H3)	AL157778.1	RNPS1P1 (H3)	LINC02055 (H2)	AC093916.1 (H3)
		LRFN5 (H3)		MEI4 (H3)	AL390334.1 (H2)	LINC02055 (H3)
		UGT8 (H3)		LRFN5 (H3)	LINC00534 (H2)	AC390334.1 (H3)
		DTX4 (H3)		DACH2 (H3)	KCNH8 (H2)	LINC00534 (H3)
		POTEG (H3)		UGT8 (H3)	LRFN5 (H2)	KCNH8 (H3)
		AL157778.1 (H3)		AC006525.1 (H3)	UGT8 (H2)	RNPS1P1 (H3)
		AL445072.1 (H3)		POTEG (H3)	AC006525.1 (H2)	MEI4 (H3)
		AP002001.2 (H3)		AL157778.1 (H3)	AC026414.12 (H2)	LRFN5 (H3)
		AL357139.2 (H3)		CENPI (H3)		DACH2 (H3)
		KRT19P6 (H3)		AP002001.2 (H3)		UGT8 (H3)
		PMPCAP1 (H3)		KRT19P6 (H3)		AC006525.1 (H3)
		HMGB1P38 (H3)		PMPCAP1 (H3)		POTEG (H3)
		AC026414.1 (H3)		HMGB1P38		AC157778.1 (H3)
				AC026414.1 (H3)		AL445072.1 (H3)
						AP002001.2 (H3)
						KRT19P6 (H3)
						PMPCAP1 (H3)
						HMGB1P38
						AC026414.1 (H3)

Figure S8. The higher amount immune and stromal cells in the HCC subgroups.

	HERV-H1vs HERV-H2	HERV-H1vs HERV-H3	HERV-H1vs HERV-L	HERV-H2 vs HERV-H3	HERV-H2 vs HERV-L	HERV-H3 vs HERV-L
**X-cell**	hepatocytes (H2)	CD8+ Tcm (H1)	hepatocytes (L)	hepatocytes (H2)		ly endothelial cells (L)
	adipocytes (H2)		adipocytes (L)	HSC (H2)		megakaryocytes (L)
	plasma cells (H2)		plasma cells (H2)	ly endothelial cells (H2)		basophil (H3)
	mast cells (H1)		mast cells (H1)	endothelial cells (H2)		Th2 cells (H3)
	mesangial cells (H1)			pDC (H2)		
				megakaryocytes (H2)		

Figure S9. The up- and down-expression genes in the BS008 treatment for the gene panels.

HERV activation restriction (96)		KZFPs (101)		RNA transport (70)		Stemness (126)		Metabolism (51)		Antivival immunity (263)		HLA&antigen presentation (45)		Immune checkpoint (22)		Inflammasome and Inflammatory response (54)		SPARCS (15)		Nearby genes (262)	
Up	Down	Up	Down	Up	Down	Up	Down	Up	Down	Up	Down	Up	Down	Up	Down	Up	Down	Up	Down	Up	Down
APOBEC3H	KDM1A	ZNF263	ZNF132	CDKN2B	ZC3H11A	CCL26	PROM1	PCK2	OTC	ATG5	CD38	ERAP2	TAPBP	VSIR	TNFRSF9	CXCL2	IL4R	TRIM38	TNFRSF9	ZNF702P	DLEU1
KLF5	YTHDC2	ZNF416	ZKSCAN5	SRSF12	NDC1	RIOK2	MTREX	SLC1A2	ACSM2B	NFKB2	MAPK9	TAP1	HLA-Z	CD160	LAG3	NLRC4	PYCARD	ADAM19	ANTXR1	GABRA2	PRDM7
BAZ2B	MBD3	ZNF213	ZNF441	DDX19B	NUP37	MTHFD2	ENTPD2	SLCO1B3	PKM	CYLD	EIF2AK2	HLA-DMA	HLA-DOB	CD70	CD276	NFKBIA	IL1R2	HERC3	SERPINB9	GRIA4	ACSS3
AGO2	ATRX	ZNF419	ZNF248	TAP1	NUP188	FST	TRNT1	XDH	ENO1	SIGLEC1	EDN1	HLA-E		HAVCR2	CD96	MMP9	IL1R1	BEND6		LMNTD1	AC098828.1
APOBEC2	DNMT3B	ZNF549	ZNF534	DDX39B	SRSF5	GNL2	SEMA3A	CES5A	MTHFD1	GADD45B	HSP90AA1	HLA-F		CD27	CTLA4	TIMP1	IL18R1			ZNF221	AC019197.1
AGO4	ALKBH5	ZNF211	ZNF736	SARNP	NUP107	HTR7	BCKDHB		AQP9	NFKBIA	IRAK3	HLA-F-AS1		BTLA		IL7	TLR2			ZNF678	AC027613.1
SETDB2	PCIF1	ZNF45		SRSF8	NUP155	HDX	DNMT3B		SULT2A1	RNF125	DDX17	HLA-K				DDX58	CXCL5			AC005487.1	LINC01060
MORC3	SUV39H1	ZNF133			SRSF4	ALX1	SNRPD3		SC5D	IKBKB	EP300	HLA-T				IL1RL1	NOD2			MIR4500HG	AC109439.1
TET2	EZH2	ZNF484			NUP43	ARMCX2	ATP11C		ACSS3	NFKBIB	TLR8	HLA-L				IL15RA	MYD88			AL591501.1	LINC02055
PIWIL2	TRDMT1	ZNF557			THOC2	TBCE	CENPI		SLC6A12	RELB	CD276					IL1RN	TLR6			LUCAT1	AC010378.1
	FOXM1	ZNF426			NUP214		ERCC2		ALDH5A1	TFEC	ZC3HAV1					NLRC5	TLR7			AC104596.1	AC093746.1
	HDAC1	ZNF304			THOC6		BMPR1A		ACADL	TAX1BP1	LGALS3BP					CXCL3	CCL5			AC079160.1	AP003327.2
	ARID1A	ZIM3			NUP210		DHX15		KMO	SERPINE1	MAPK10					NLRC3				AC244502.1	AL162493.1
	DNMT3A	ZNF614			NUP42		KIF20A		ALDH6A1	DDX58	DHX15					IL7R				LINC02163	AC010328.1
	HNRNPA2B1	RBAK			SRSF1		GBE1		CYP2C8	CXCL12	CAMKK2					CXCL10				AL390334.1	MGAT4A
	SMUG1	ZNF222			NCBP1		MSH6		SORD	DHX58	OAS3									KCNH8	CCRL2
	PIWIL1	ZNF333			NUP205		ICMT		CYP11A1	HBEGF	MAPK14									HNRNPA3P8	AC026444.1
	APOBEC3A	ZNF577			SRSF2		TRIM24		SLC13A5	GBP3	GLS									RNPS1P1	CHSY3
	DNMT1	ZNF496			POM121		DLGAP5		TRPM2	GBP1	TRIM32									SH3RF1	MEI4
	MBD2	ZNF202			NUP62		PAICS		GLYAT	TNFAIP3	IFIT3									AL353133.1	AC079804.3
	PPHLN1	ZNF597					HAT1		MAT1A	IFIT2	CCRL2									CCN6	TRIM24
	PIWIL4	ZNF212					MYCN		CYP3A4	NT5C3A	TNFSF10									AC087343.1	AP001979.1
	IGF2BP3	ZNF160					NREP		NAGS	NR4A1	RBBP6									LINC02761	MED15P6
	TET1	ZNF561					TET1			TREM1	PIN1									MAOA	AL079352.1
	SUV39H2	ZNF274					INHBE			IRF1	NECTIN2									HNRNPCP10	BNIP3P1
	IGF2BP1	ZNF354C					HMGA2			TNFSF14	SHFL									LINC02611	LRFN5
	CHAF1B	ZNF543					NMNAT2			IL15RA	MAP3K7									ELOAP1	Z70280.1
	TASOR	ZNF223					MIS18A			STX11	DRAM1									AC006460.2	PLS3-AS1
	CHAF1A	ZNF571					IGF2BP1			PIM1	RTP4									HMGB1P38	AC092170.1
	APOBEC3B	ZNF746					HESX1			IRF4	IFI44									CD200R1L	HS3ST1
	SOX2	ZNF93					ANPEP			DDX60	PARP9									AC034229.1	HNRNPA1P67
	MBD5	ZNF566					TBC1D16			PHLDA1	LY96									AC122694.1	PMPCAP1
	APOBEC3D	ZNF749					XXYLT1			NLRC5	LY6E									PTP4A1P4	UGT8
	APOBEC3C	ZNF197					FZD2			TRIM4	CTLA4									AC006525.1	ARL2BPP4
	RBM15B	ZNF793					ROR1			PTGES	ABCA1									LINC02527	NMBR
	UHRF1	ZNF567					IARS1			CDC42EP2	FADD									IGSF9B	SLC22A3
		ZNF79					ADH5			UBE2L6	RNASE2									C17orf58	AC090796.1
		ZKSCAN7					TBCE			MAPK13	OLR1									RPL7P2	AP002001.2
		ZNF136								ETS2	SAA1									MTCO1P53	CENPI
		ZFP28								MX1	MARCKSL1										AL627231.1
		ZNF763								GBP2	TBK1										
		ZNF772								ATF3	STING1										
		ZNF649								REL	IRF7										
		ZKSCAN8								AZI2	PLSCR1										
		ZNF442								TLR3	HMGB1										
		ZNF808								IFI27	TLR7										
		ZNF627								TAP1	DDX3X										
		ZNF611								IL12A	TAPBP										
		ZNF812P								CXCL10	IKBKE										
		ZNF850								CXCL8	CCL5										
		ZNF2								STAT2											
										ISG20											
										TRAF6											
										SNX18											
										RNF135											
										SOCS3											
										MAFF											
										MAPK11											
										TRIM69											
										ISG15											
										TXNRD1											
										GK											
										AGER											
										GBP1P1											
										TNF											
										PSMB9											
										TICAM2											
